# Perceived importance of moderate‐to‐vigorous physical activity as a weight control strategy in behavioral weight loss

**DOI:** 10.1002/osp4.695

**Published:** 2023-07-08

**Authors:** Marny M. Ehmann, Erica M. LaFata, Hannah C. McCausland, Francesca M. Knudsen, Meghan L. Butryn

**Affiliations:** ^1^ Department of Psychological and Brain Sciences and Center for Weight Eating and Lifestyle Science (WELL Center) Drexel University Philadelphia Pennsylvania USA

**Keywords:** behavioral weight loss, moderate‐to‐vigorous physical activity, obesity, overweight, weight control strategies

## Abstract

**Background:**

Previous research has established the importance of moderate‐to‐vigorous physical activity (MVPA) for weight control. One area of unexplored investigation is the relationship between individuals' perceptions of the importance of MVPA for weight control and MVPA engagement. This study examined the associations between the perceived importance of MVPA and MVPA engagement, weight loss, barriers to PA, and exercise enjoyment in adults enrolled in a long‐term behavioral weight loss (BWL) intervention.

**Methods:**

Adults (*N* = 301) with overweight/obesity (BMI = 27–45 kg/m^2^) completed an 18‐month BWL intervention, followed by a no‐intervention 18‐month follow‐up. At baseline, 6 months, 18 months (i.e., post‐treatment), and 36 months (i.e., follow‐up), participants ranked the importance of six strategies for weight control: keeping a food record, MVPA, light PA, self‐weighing, small portions, and low‐calorie diet. Observed MVPA (measured by accelerometer), percent weight loss, perceived barriers to PA, and exercise enjoyment were also measured at each assessment.

**Results:**

Results showed that most participants perceived MVPA as a primary weight control strategy (first, second, or third most important) throughout the intervention, regardless of the weight control goal (weight loss vs. maintenance). Individuals who ranked MVPA as a primary strategy for weight control at concurrent time points, compared to those who did not, engaged in significantly more MVPA at post‐treatment, had greater weight loss at follow‐up, endorsed fewer barriers to PA at post‐treatment and follow‐up, and reported greater exercise enjoyment at baseline and post‐treatment.

**Conclusion:**

Perceived importance of MVPA was related to subjective experiences of MVPA, MVPA adherence, and weight loss in a long‐term BWL intervention.

## Graphical abstract

One area of unexplored investigation is the relationship between individuals' perceptions of the importance of moderate‐to‐vigorous physical activity (MVPA) for weight control and physical activity engagement. This study examined the associations between the perceived importance of MVPA and physical activity engagement, weight loss, barriers to physical activity, and exercise enjoyment in adults enrolled in a long‐term behavioral weight loss intervention. Results showed that most participants perceived MVPA as a primary weight control strategy (first, second, or third most important) throughout intervention, regardless of weight control goal (weight loss vs. maintenance), and that perceived importance of MVPA was related to subjective experiences of physical activity, physical activity adherence, and weight loss.

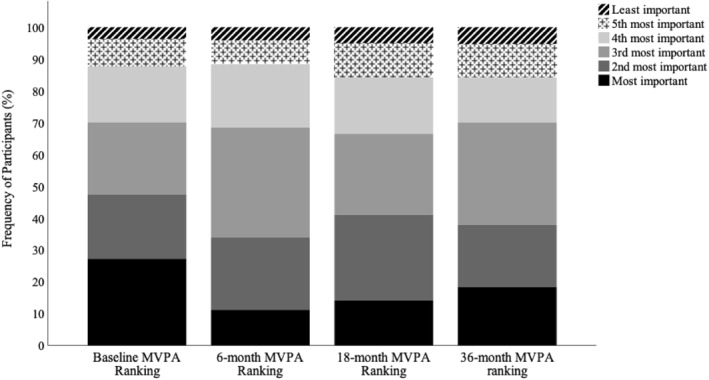

## 1 INTRODUCTION

With the prevalence of overweight and obesity in the U.S. approaching 74%, interventions that facilitate lifestyle modification are essential.^1^ Multicomponent behavioral weight loss (BWL) interventions teach participants strategies for long‐term weight loss, including reducing calorie intake, engaging in behavioral skills (e.g., self‐monitoring, goal‐setting, stimulus control), and adopting moderate‐to‐vigorous physical activity (MVPA).^2^ Engaging in high amounts of MVPA (e.g., 200–300 min/week) facilitates weight loss, reduces the likelihood of weight regain following losses,^3^ and produces health benefits (e.g., reduced risk for diseases).^4^, ^5^, ^6^ Although activity energy expenditure has generally increased since the 1980s in the U.S.^7^ and BWL is an evidence‐based obesity treatment, adherence to MVPA recommendations is poor: many participants in BWL programs do not adopt or maintain prescribed amounts of MVPA throughout or beyond treatment.^8^, ^9^, ^10^

A large body of research has been conducted to understand the MVPA adherence gap.^11^, ^12^, ^13^, ^14^, ^15^, ^16^ One factor that has yet to be evaluated rigorously is participants' beliefs about the role of MVPA in long‐term weight control. According to the theory of planned behavior, favorable evaluations about the importance of MVPA for weight control may impact MVPA engagement and weight loss by enhancing intentions to engage in MVPA behavior.^17^, ^18^ If participants do not anticipate benefit or have unfavorable attitudes toward MVPA engagement, particularly relative to other weight control behaviors, this may lead to poor adherence. It is also possible that poor MVPA engagement itself reduces the perceived importance of MVPA, as individuals may discount the importance of behaviors they struggle to engage in.^19^ We know of no previous studies that have tested whether perceived importance of MVPA is related to MVPA engagement or weight loss in a long‐term BWL sample.

There is also limited research examining how the perception of the importance of MVPA relates to subjective experiences of MVPA. Previous research has found that greater exercise enjoyment and fewer perceived barriers to PA (e.g., lack of time, skill) are associated with greater MVPA engagement, suggesting that these are important determinants of exercise behavior.^20^, ^21^ It may be that participants who experience many barriers to MVPA or do not enjoy MVPA come to believe that it is relatively unimportant for weight control because they wish to be successful at weight loss regardless of adherence to a behavioral goal that they perceive as challenging or unpleasant.

It is also unknown whether participants' weight control goals (weight loss vs. weight loss maintenance) impact their views about the importance of MVPA. Intervention staff typically educate participants that MVPA is a less powerful driver of weight change during the initial phase of weight loss (e.g., months 1–6), but is an influential determinant of weight loss maintenance.^2^, ^3^ Some studies have assessed participants' beliefs about strategies important for weight loss and maintenance,^22^, ^23^, ^24^, ^25^ but no studies have analyzed differences in the perceived importance of weight control strategies for those attempting weight loss versus weight loss maintenance. Understanding the correspondence between MVPA perceptions and weight control goal might help explain high rates of weight regain during the weight loss maintenance phase.

To our knowledge, this is the first study to investigate perceived importance of MVPA as a weight control strategy in a long‐term, randomized controlled trial (RCT) of BWL for adults with overweight/obesity. Aim 1 characterized the perceived importance of MVPA relative to other weight control strategies (e.g., keeping a food record) at baseline and examined changes in the perceived importance of MVPA throughout BWL intervention (i.e., baseline vs. 6 and 18 months) and follow‐up (baseline vs. 36 months). Aim 2 examined the associations between the perceived importance of MVPA and objectively measured MVPA engagement, weight loss, perceived barriers to PA, and exercise enjoyment at baseline, post‐treatment (18 months), and follow‐up (36 months). Aim 3 assessed the relationship between the perceived importance of MVPA and the stated weight control goal (weight loss vs. weight loss maintenance) at post‐treatment (18 months) and follow‐up (36 months). Our hypotheses were as follows: (1) the perceived importance of MVPA would significantly differ from other weight control strategies at baseline and significantly change over the course of BWL intervention; (2) individuals who ranked MVPA as a primary weight control strategy would engage in more MVPA, have greater weight loss, report fewer barriers to PA, and endorse higher exercise enjoyment during BWL intervention; and (3) the perceived importance of MVPA would be higher in those pursuing weight loss maintenance compared to those attempting to lose weight.

## 2 METHOD

### 2.1 Participants

This project was a secondary analysis of data collected from participants recruited in the Philadelphia area between 2014 and 2016 for an RCT of BWL.^26^ Eligible participants had a BMI between 27 and 45 kg/m^2^, were 18–70 years old, were physically able to exercise (i.e., walk at least 2 blocks without stopping), and completed enrollment procedures (i.e., attendance at eligibility sessions and completion of 3‐day food record). Exclusion criteria included medical or psychiatric conditions that would pose a risk to the participant or cause a weight change during the program, current or expected pregnancy or breastfeeding, recent start or changes in medication that could impact weight, bariatric surgery history, and weight loss of ≥5% in the previous 6 months. All enrolled participants provided informed consent and were compensated for participation.

### 2.2 Procedures

Detailed study procedures for the parent study were reported elsewhere.^26^, ^27^ Briefly, the parent study included two phases of BWL treatment. In Phase I, all participants received 6 months (16, 75‐min sessions) of group‐based standard BWL treatment including skills adapted from LOOK AHEAD and the Diabetes Prevention Program (e.g., self‐monitoring of calorie intake, goal‐setting).^28^, ^29^ Consistent with recommendations for MVPA for weight loss and weight loss maintenance from literature,^2^, ^3^ participants were encouraged to self‐monitor and gradually increase MVPA over the first 6 months of the program, with the goal of reaching and maintaining 250 min per week. Participants self‐reported weekly MVPA minutes in each group session to enhance accountability and problem solve as needed. In the standard BWL condition, 65%, 25%, and 10% of the session time focused on eating behavior, PA, and other behaviors (e.g., self‐monitoring of weight), respectively. In Phase II (months 7–18), participants completed 14 group sessions and three 15‐min coach calls in one of three randomly assigned groups: standard BWL from Phase I (i.e., identical to what all participants received in Phase I), BWL with an emphasis on PA, or acceptance‐based BWL with an emphasis on PA. The BWL with an emphasis on PA condition included the same behavioral skills as the standard BWL condition (e.g., goal‐setting, self‐monitoring) but sessions emphasized meeting PA goals (i.e., 65% of session focused on PA, 25% on eating behaviors, and 10% on other). In the acceptance‐based BWL with an emphasis on PA condition, sessions emphasized meeting PA goals (i.e., 65% of session focused on PA, 25% on eating behaviors, and 10% on other) and skill‐building components were taught from an acceptance‐based framework (e.g., non‐judgmental acceptance toward MVPA).^30^ Post‐treatment represented month 18 of the program, which was the end of the intervention period. Months 19–36 was a no‐treatment follow‐up period. Assessment measures for the parent study were completed at baseline, 6, 12, 18, 24, and 36 months. The current study analyzed outcomes at baseline, 6 months, 18 months, and 36 months.

### 2.3 Measures

#### 2.3.1 Demographics

Participants self‐reported age, gender, race, and ethnicity at baseline.

#### 2.3.2 Weight control strategies

At each assessment, participants completed an investigator‐derived measure in which they indicated their current weight control goal (i.e., weight loss or weight loss maintenance) and ranked ordered the importance (1 = most important to 6 = least important) of six weight control strategies in reaching said goal: keeping a food record, low‐calorie diet, MVPA, weighing self regularly, light PA, and eating small portions.

#### 2.3.3 Weight

Body weight was measured by a blinded research staff at each assessment using a Tanita ® model WB‐3000 digital scale. This scale was accurate to 0.1 kg. Two measurements were taken and averaged for the final measurement. Weight loss was calculated as the percentage of weight lost from baseline to the assessment point.

#### 2.3.4 MVPA

MVPA was calculated using waist‐worn ActiGraph (Pensacola, FL) GT3X tri‐axial solid‐state accelerometers. Participants were instructed to wear devices for all waking hours on 7 consecutive days at assessments. Data were considered valid and included in analyses if the participant wore the device for a minimum of 4 days for 10 or more hours per day. ActiLife software was used to extract and calculate MVPA in bouts of at least 10 min with established cutoff points.^31^ Total MVPA (minutes/week) and achievement (≥250 min/week) versus non‐achievement (<250 min/week) of MVPA program recommendations were calculated.

#### 2.3.5 Barriers to physical activity

Barriers to PA were assessed using the Barriers to Being Active Quiz, a 21‐item measure assessing seven barriers to PA (e.g., lack of time, social influence, lack of energy) that has demonstrated high reliability in previous studies.^32^, ^33^ Participants rated each statement on a scale from 0 (very unlikely) to 3 (very likely). Scores were summed (possible range = 0–63), with higher scores indicating greater perceived barriers to PA.

#### 2.3.6 Exercise enjoyment

Exercise enjoyment was measured using the Physical Activity Enjoyment Scale (PACES), a reliable and valid 16‐item measure in which participants rated statements about exercise (e.g., “I enjoy it”) from 1 (disagree a lot) to 5 (agree a lot).^34^ Items in the opposite reaction (e.g., “I dislike it”) were reverse scored and averaged. Higher mean PACES scores indicated greater exercise enjoyment.

### 2.4 Data analysis

Data were analyzed in SPSS version 28.0.^35^ At baseline, 301 participants (out of 320 in the parent study) completed the weight control strategies measure. Throughout the study, three participants were withdrawn by the investigator, 46 participants dropped during Phase 1 (baseline to 6 months), and 21 dropped during Phase II (months 7–18). Those who dropped from the study at any point in time did not differ from those who remained in the study by race, baseline MVPA, or baseline perceived importance of MVPA (*p*s > 0.05). Those who dropped were significantly younger (*M* = 49.1, *SD* = 11.6) and had a higher BMI at baseline (*M* = 36.4, *SD* = 4.8). Available data for the weight control strategies measure was 80% at 6 months (*n* = 241), 65.4% at 18 months (*n* = 197), and 63.1% at 36 months (*n* = 190). Analyses were run at a significance level of 0.05 with available data at each time point. All reported *p* values were based on two‐sided hypotheses, except when directional hypotheses warranted one‐sided investigation. In aims that analyzed perceived importance of MVPA as the outcome variable, non‐parametric statistical alternatives were used to account for an ordinal data scale. MVPA ranking was transformed into a dichotomized grouping variable of those who ranked MVPA as a primary strategy (i.e., within the top three strategies), versus those who did not (i.e., bottom three strategies), to assess the differences in health behaviors between these two groups.

Participant characteristics were measured using descriptive statistics. A Friedman test compared whether the rank order of the six weight control strategies significantly differed prior to intervention and Wilcoxon‐signed rank tests assessed whether the perceived importance of MVPA changed from baseline to 6 months (*n* = 230), 18 months (*n* = 188), and 36 months (*n* = 183). Wilcoxon‐signed rank tests were used instead of a Friedman ANOVA to avoid high rates of listwise deletion and to include relevant pre‐treatment, post‐treatment, and follow‐up assessments. Independent samples *t*‐tests were used to assess the concurrent differences in MVPA (average min/week), weight loss, barriers to PA, and exercise enjoyment between those who ranked MVPA as a primary weight control strategy and those who did not at baseline, 18 months (post‐treatment), and 36 months (follow‐up). Chi‐square tests for independence also examined whether perceived MVPA importance was significantly related to the achievement of program recommendations for MVPA (≥250 min/week). Concurrent relationships were examined given evidence from the theory of planned behavior that specific behaviors can be predicted from attitudes if the measures are compatible, meaning that they involve the same target, act, context, and time.^19^ To be consistent with these criteria, especially time, the relationships between current MVPA importance and current MVPA behaviors and subjective experiences of MVPA were examined. The pre‐treatment, post‐treatment, and follow‐up time points were selected to reduce overall analyses and capture relevant treatment periods in BWL intervention. Mann Whitney U tests were performed to compare whether rank ordered MVPA importance differed between those wanting to lose weight or maintain weight loss at months 18 and 36 (<4% of the sample in weight loss maintenance at baseline and 6 months).

### 2.5 Ethical approval

The parent study was registered with ClinicalTrials.gov (NCT02363010) and was approved by the Institutional Review Board of Drexel University.

## 3 RESULTS

### 3.1 Sample characteristics

The sample (*N* = 301) was 78% female with a mean age of 52.8 years (*SD* = 10.3) and an average BMI of 35.1 kg/m^2^ (*SD* = 4.8) at baseline. The racial composition of the sample was 71.4% White, 23.9% Black/African American, 1.3% Asian, 0.3% Native American/Alaskan Native, and 3.0% multi‐racial. Four percent of the participants were Hispanic/Latinx. At baseline, participants engaged in an average of 59.3 min per week (*SD* = 83.0) of MVPA. At the end of treatment (18 months), participants achieved an average weight loss of 10.8% (*SD* = 10.7) and average MVPA of 137.65 min per week (*SD* = 128.53). Independent samples *t*‐tests revealed no significant differences in age and BMI between those who classified MVPA as a primary weight control strategy and those who did not at baseline (all *p*s > 0.79). Chi‐square tests for independence demonstrated no significant relationships between gender or race and perceived MVPA importance at baseline (*p*s > 0.22). Behavioral weight loss experimental conditions were collapsed for analyses given that endorsement of MVPA as a primary weight control strategy was not significantly related to treatment condition (*p*s > 0.18) and treatment conditions did not significantly differ in weight loss or MVPA at post‐treatment (18 months) or follow‐up (36 months).^26^, ^27^

### 3.2 Perceived MVPA importance at baseline and across behavioral weight loss intervention

**FIGURE 1:**
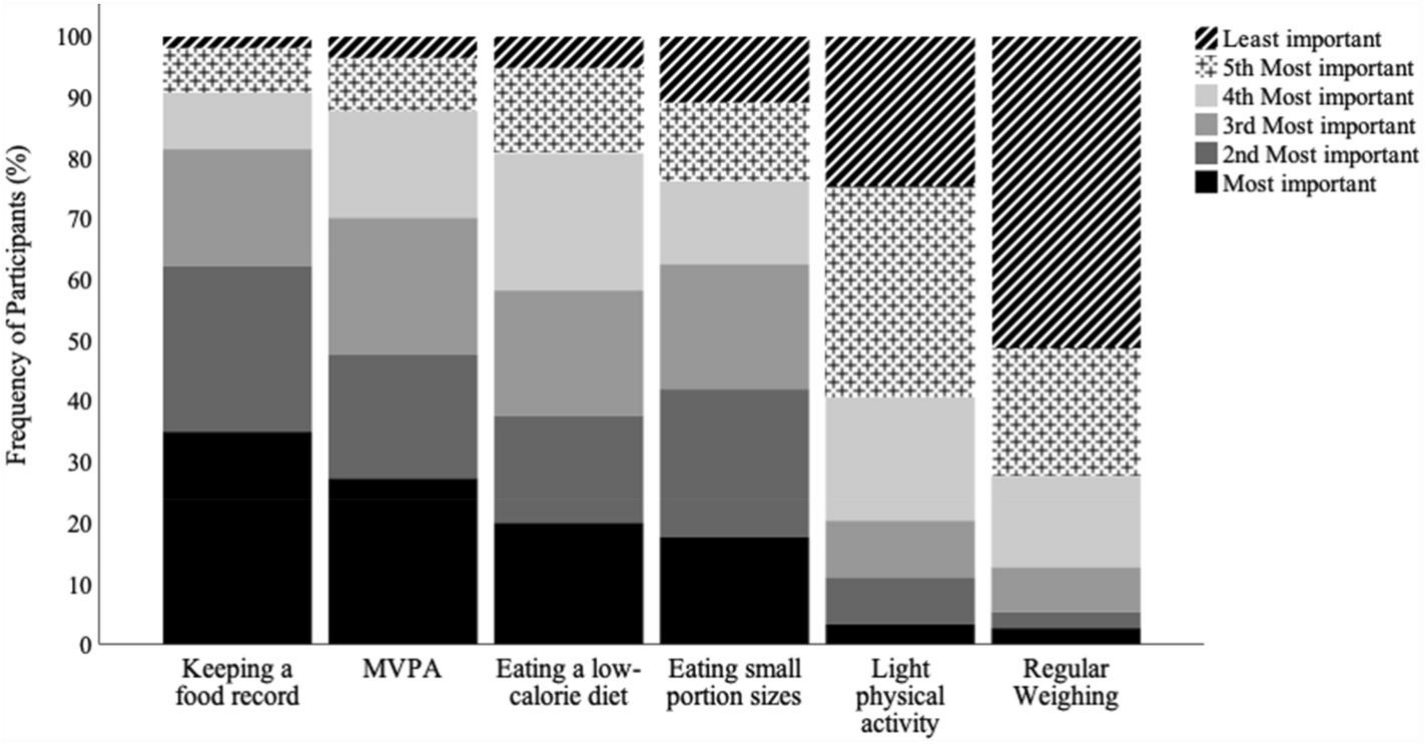
Perceived importance of weight control strategies at baseline.

Figure 1 illustrates the perceived importance of each weight control strategy at baseline. A Friedman test revealed that participants ranked weight control strategies as differentially important at baseline, *χ* ^2^(5) = 489.83, *p* < 0.001, *w* = 0.33. The order of most important (i.e., lower mean rank) to least important strategy was keeping a food record (*M* = 2.33), MVPA (*M* = 2.71), eating a low‐calorie diet (*M* = 3.09), eating small portions (*M* = 3.13), light PA (*M* = 4.50), and regular self‐weighing (*M* = 5.03). Post‐hoc pairwise comparisons with Bonferroni corrections indicated that MVPA was ranked significantly more important than regular self‐weighing and light PA (*p*s < 0.001), but did not significantly differ from keeping a food record, eating a low‐calorie diet, or eating small portions (*ps* ≥ 0.08).

**FIGURE 2:**
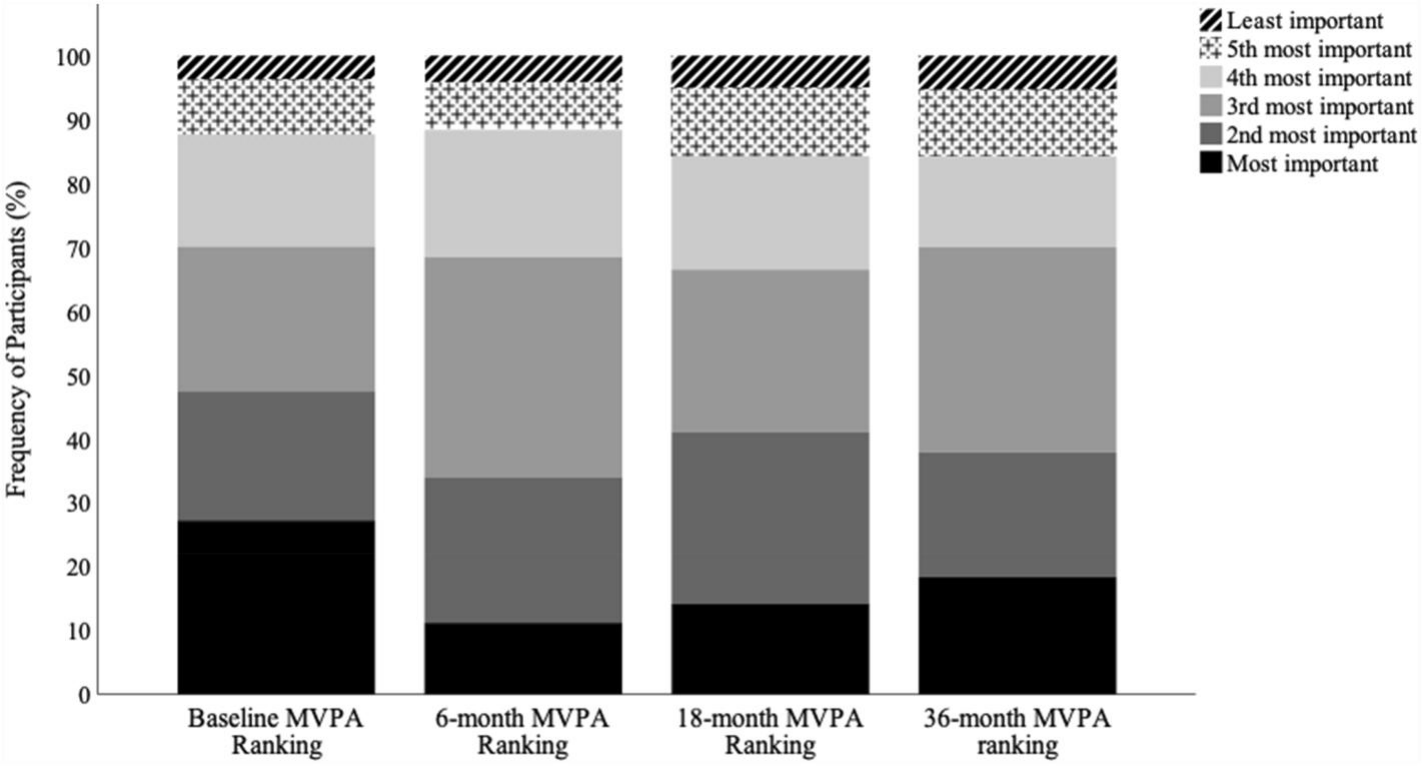
Changes over time in perceived importance of MVPA.

Figure 2 illustrates the change in perceived MVPA importance throughout BWL. Wilcoxon‐signed rank tests showed that participants ranked MVPA as significantly less important for weight control at 6 months compared to baseline, *Z* = −2.93, *p* = 0.003, *r* = 0.13, but no difference from baseline at 18 and 36 months (*p*s ≥ 0.08).

### 3.3 Associations between perceived importance of MVPA and health behaviors

Table 1 presents the means, standard deviations, and independent samples *t*‐test results for MVPA behaviors between those who ranked MVPA as a primary strategy and those who did not. Independent samples *t*‐tests revealed that those who ranked MVPA as a primary weight control strategy (ranging from 64.7% to 71.4% of the sample during the study), compared to those who did not (ranging from 28.6% to 35.3% of the sample during the study) at concurrent time points, engaged in significantly more minutes of MVPA at 18 months (*t*(139) = 2.19, *p* = 0.015, *d* = 0.40), endorsed significantly fewer barriers to PA at 18 (*t*(175) = 2.25, *p* = 0.013, *d* = 0.36) and 36 months (*t*(138) = 1.92, *p* = 0.028, *d* = 0.36), indicated higher exercise enjoyment at baseline (*t*(298) = 3.20, *p* < 0.001, *d* = 0.40) and 18 months (*t*(98.8) = 2.29, *p* = 0.012, *d* = 0.39), and had greater mean weight loss at 36 months (*t*(118.15) = 1.98, *p* = 0.03, *d* = 0.30).

**TABLE 1:** Independent samples *t*‐tests of differences in outcomes between those who ranked MVPA as a primary weight control strategy (Primary) and those who did not (Not Primary).

		Primary	Not Primary	*t*	*d*
		*M (SD)*	*M (SD)*		
MVPA (min/week)	Baseline (*n* = 293)	59.5 (86.7)	58.5 (73.7)	0.10	0.01
	6 months (*n* = 223)	147.1 (120.6)	128.5 (127.1)	1.04	0.15
	18 months (*n* = 141)	155.4 (141.7)	104.4 (95.4)	2.19*	0.40
	36 months (*n* = 134)	117.2 (121.5)	98.5 (93.9)	0.87	0.16
Weight loss (%)^a^	6 months (*n* = 238)	10.5 (5.1)	10.0 (5.1)	0.65	0.09
	18 months (*n* = 192)	11.4 (12.0)	10.6 (10.1)	0.50	0.08
	36 months (*n* = 162)	6.6 (9.1)	4.1 (6.4)	1.98*	0.30
Barriers to PA	Baseline (*n* = 277)	17.2 (9.4)	18.6 (9.8)	1.14	0.15
	6 months (*n* = 215)	13.3 (10.8)	13.9 (9.7)	0.38	0.06
	18 months (*n* = 177)	12.3 (10.3)	15.9 (9.8)	2.25*	0.36
	36 months (*n* = 140)	13.7 (10.3)	17.5 (11.8)	1.92*	0.36
Exercise enjoyment	Baseline (*n* = 301)	4.1 (0.6)	3.9 (0.7)	3.20***	0.40
	6 months (*n* = 241)	4.1 (0.6)	4.0 (0.8)	1.05	0.15
	18 months (*n* = 197)	4.1 (0.7)	3.8 (0.9)	2.29*	0.39
	36 months (*n* = 190)	4.2 (0.7)	4.0 (0.8)	1.22	0.21

^a^ Higher values indicate greater weight loss (%). Weight loss (%) was measured from baseline to the time point.

*p < 0.05, ***p < 0.001.

Regarding the achievement of program recommendations for MVPA, only the minority of the sample achieved ≥250 min/week of MVPA at each assessment point: 3.2% (*n* = 10) at baseline; 18.1% (*n* = 48) at 6 months; 17.5% (*n* = 30) at 18 months; and 16.7% (*n* = 28) at 36 months. Perceiving MVPA as a primary weight control strategy was not significantly related to the achievement of program recommendations for MVPA (*p*s ≥ 0.22).

### 3.4 Relationship between weight control goal and perceived importance of MVPA

Results from Mann‐Whitney U tests indicated no significant differences in perceived MVPA importance between those with a weight loss goal (71.6% at post‐treatment and 87.9% at follow‐up) and those with a maintenance goal (28.4% at post‐treatment and 12.1% at follow‐up) at any assessment point (all *p*s ≥ 0.23).

## 4 DISCUSSION

To our knowledge, this was the first study to assess the perceived importance of MVPA as a weight control strategy throughout a long‐term BWL intervention. Because most individuals fail to meet the prescribed MVPA for weight control, it is integral to understand whether the perceived importance of MVPA may have implications for adherence rates.^9^ The findings from the present study suggested that beliefs about the importance of MVPA may decrease slightly as individuals learn about the unique contributions of MVPA and dietary changes to weight loss, but ultimately remain high relative to other strategies across a weight control effort. The majority of participants perceived MVPA to be one of the most important weight control strategies prior to and throughout BWL intervention, regardless of weight control goal (i.e., loss vs. maintenance). However, the minority who ranked MVPA as less important for weight control compared to other strategies engaged in fewer minutes of MVPA at post‐treatment, had lower weight loss at follow‐up, perceived more barriers to MVPA at post‐treatment and follow‐up, and reported less exercise enjoyment at baseline and post‐treatment than those who perceived MVPA as a primary strategy, suggesting that individuals' perceptions are linked to important treatment outcomes. There may be bidirectional relationships between the perceived importance of MVPA, subjective experiences of MVPA, and adherence to this behavior.

Results suggested that MVPA perceptions were moderately consistent throughout BWL intervention and across changing weight control goals. Participants ranked MVPA as the second most important weight control strategy prior to intervention, and MVPA perceptions changed only slightly throughout BWL treatment. MVPA was ranked as significantly less important for weight control at 6 months compared to baseline, but not different from baseline at 18 and 36 months. The decrease in the perceived importance of MVPA at 6 months relative to other weight control strategies (e.g., ranking MVPA as most important at baseline and second or third most important strategy at 6 months), aligns with the prominent focus on eating behavior during the first 6 months of standard BWL education.^2^ Furthermore, the perceived MVPA importance did not differ by weight control goal, despite ample BWL education regarding the importance of MVPA for weight loss maintenance.^36^, ^37^ This relative stability in attitudes toward MVPA may suggest that BWL education does not effectively help change the valuation of MVPA when participants enter the weight loss maintenance phase. It could also be that previous experiences shaped participant perceptions more than BWL education,^38^, ^39^ or that there was a ceiling effect preventing statistically significant increases in importance during the weight loss maintenance phase, given that the perceived importance of MVPA was high from the start of the study.

Despite the relative stability of MVPA perceptions, results showed that the perceived importance of MVPA is related to MVPA engagement and facilitators. Those who ranked MVPA as a primary weight control strategy engaged in more MVPA at post‐treatment, had higher weight loss at follow‐up, endorsed fewer barriers to PA at post‐treatment and follow‐up, and reported higher exercise enjoyment baseline and post‐treatment than those who did not. There were no significant differences in MVPA, BMI, or perceived barriers to PA at baseline, likely due to participants not yet being engaged in their weight control effort. In accordance with the theory of planned behavior, these findings suggest that perceiving MVPA as important for weight control may be one influential factor that makes individuals more likely to report positive subjective experiences of MVPA and engage in MVPA,^17^ while undervaluing the importance of MVPA is a cognitive barrier to engaging in MVPA for weight control. This should be considered in the context of other objective weight control behaviors that individuals engage in.

Regarding observed MVPA, results indicated that the perception that MVPA was important for weight control corresponded to greater MVPA engagement at post‐treatment (18 months), representing a potential link between attitudes toward MVPA and behavioral engagement.^17^, ^40^ Health‐promoting intention‐behavior research has recognized the importance of pre‐intentional motivation factors and post‐intentional action factors that bridge the intention‐behavior gap.^41^, ^42^ In the relationship between MVPA perceptions and observed MPVA, the perceived importance of MVPA could act as pre‐intentional motivation that is mediated or moderated by relevant post‐intentional factors, such as self‐efficacy. Future research is warranted to determine whether education regarding the importance of MVPA enhances MVPA engagement via relevant post‐intentional factors. However, perceiving MVPA as more important for weight control did not relate to the achievement of program recommendations for MVPA (≥250 min/week). Furthermore, MVPA was higher in individuals who perceived MVPA as a primary weight control strategy at follow‐up (36 months) but did not reach the threshold of significance. Both of these findings are unsurprising, given that individuals often have poor adherence to MVPA recommendations in BWL interventions,^43^ especially over time and with less accountability.^44^ Although perceived MVPA importance may be one factor encouraging MVPA engagement, the constellation of perceptions and other factors known to motivate exercise (e.g., perceived control, skill) may be needed to meet program adherence goals.^45^

Furthermore, those who perceived MVPA as more important also had greater weight loss at the 36‐month follow‐up. Research has shown that individuals may fail to maintain weight loss following BWL treatment due to numerous factors, including poor MVPA engagement.^46^ Although the difference in MVPA engagement between those who endorsed MVPA as a primary weight control strategy and those who did not at 36 months was not statistically significant, it was in the same direction as 18 months (i.e., more minutes of MVPA in those perceiving MVPA as a primary strategy), suggesting a possible clinically significant difference that helped prevent weight regains. Future research is warranted to investigate the link between MVPA perceptions and behavior using tools that continuously measure MVPA (e.g., wearable trackers) and weight (e.g., daily weighing using smart scales) to better parse out these relationships. It could also be helpful to examine eating behaviors in conjunction with MVPA as they play a large role in the energy balance that contributes to weight control.^47^

Additionally, those who perceived MVPA as a primary weight control strategy endorsed fewer perceived barriers to PA at post‐treatment and follow‐up (18 and 36 months). Research has shown that barriers to PA predict willingness to engage in MVPA, MVPA engagement, and odds of successful weight loss.^20^, ^48^ The relationship between beliefs about the importance of MVPA and barriers to PA may be bidirectional. It could be that those who perceive MVPA as more important for weight control have an elevated sense of self‐efficacy, related to a belief that they can overcome constraints, which reduces their perception of barriers or promotes the use of problem‐solving strategies to mitigate barriers.^49^ In the opposite direction of effect, in line with cognitive dissonance and effort justification theories, individuals who experience high barriers to MVPA may reconcile dissonant cognitions about barriers and MVPA benefits by discounting the importance of MVPA, especially if they perceive low subjective reward of MVPA compared to the effort to overcome barriers to engagement.^50^

Finally, those who perceived MVPA as a primary weight control strategy endorsed greater exercise enjoyment at baseline and post‐treatment (18 months). Exercise enjoyment has been shown to be associated with intrinsic motivation for exercise and BWL intervention dropout risk.^51^, ^52^ It may be that appraising MVPA as highly important enhances the enjoyment of MVPA, as suggested by the control value theory of achievement emotions.^53^ In the opposite direction of effect, exercise enjoyment may increase the perception of the importance of MVPA, given the evidence that exercise enjoyment is related to a sense of exercise identity that assigns meaning and importance to the behavior.^54^, ^55^ Non‐significant differences in exercise enjoyment between groups at follow‐up (36 months) was due to mean increases in exercise enjoyment compared to previous time points in those who did not rank MVPA as a primary weight control strategy. This may be because these individuals were no longer participating in the active BWL trial and were no longer encouraged to follow a prescribed MVPA regimen or report on goal progress. Because of this, they may have viewed MVPA more favorably because of a decline in accountable pressure. Alternatively, these individuals could have learned to enjoy exercise more throughout the program.

Given the results that MVPA perceptions correspond to observed MVPA and MVPA adherence variables, future research is warranted to determine whether experimentally manipulating perceived importance of MVPA may directly influence MVPA engagement, long‐term weight loss, and subjective MVPA experiences. Experimentally manipulating the perceived importance of MVPA could include, but is not limited to, more targeted goal‐setting, formatting interventions to sequentially focus on eating behaviors and MVPA, and modifying clinician education to enhance supportive accountability of MVPA engagement. Clinicians may also need to be educated on biases that individuals may have toward MVPA importance and the factors contributing to negative perceptions of MVPA (e.g., weight bias internalization) to strengthen the importance of MVPA in those seeking weight loss.^56^ It would also be beneficial to examine psychosocial factors, including self‐efficacy, autonomous motivation for MVPA engagement, and social support as moderators or mediators of the associations between perceived importance of MVPA and health behaviors, given their theoretical importance in behavior engagement (e.g., self‐determination theory).^57^ Furthermore, recent evidence suggests that reductions in basal energy expenditure over time due to dietary changes (e.g., saturated fat intake) may contribute to obesity.^7^ Therefore, in conjunction with recommendations to increase MPVA, which has numerous health benefits^58^ and has been shown to be a predictor of weight loss maintenance,^59^ it would be important to consider dietary changes for optimal weight loss and maintenance. Finally, it could be beneficial to investigate this research question in studies that examine weight control perceptions, MVPA, and weight using tools (i.e., wearable trackers and smart scales) or methodologies (e.g., ecological momentary assessment) that measure variables at the daily level to clarify the variability found when measuring these variables at moments in time.

The study strengths included a long‐term investigation of weight control beliefs using a large sample of adults interested in losing weight. To our knowledge, this was the first study to use a structured self‐report questionnaire to assess the relative perceived importance of common weight control strategies. Furthermore, MVPA perceptions and outcome data were captured at numerous time points throughout the study, allowing the analysis of the link between perceptions and health behavior change throughout the intervention. Finally, MVPA was objectively measured using accelerometers compared with self‐reporting methods that may be affected by reporting bias.

One key limitation of the study was the lack of a control group who did not participate in BWL intervention, which prevents generalizability beyond BWL programs. Furthermore, most of the sample was female and White, limiting the ability to generalize results to more racially diverse groups and sexes. High variability in MVPA data may have limited the ability to detect differences that existed between the groups. Finally, similar to many longer‐term intervention studies, there was a high volume of attrition for participants lost to follow‐up.

In conclusion, the investigation of the impact of weight control perceptions on health behavior changes, relevant MVPA adherence variables, and weight loss is largely unexplored. The results of the current study suggest that the perceived importance of MVPA is associated with MVPA engagement, weight loss, barriers to PA, and exercise enjoyment throughout a long‐term BWL intervention. Future research should experimentally manipulate MVPA perceptions to ascertain causal relationships between perceptions and behaviors and establish moderation or mediation links to help address the problem of MVPA adherence in weight control.

## AUTHOR CONTRIBUTIONS

All authors conceived this project. Marny M. Ehmann, Hannah C. McCausland, and Francesca M. Knudsen conducted a literature review and drafted the introduction. Erica M. LaFata assisted in the statistical analysis plan and Erica M. LaFata and Meghan L. Butryn reviewed and approved the project in its early stages. Marny M. Ehmann wrote the original draft of the method, results, and discussion. All authors contributed to editing, reviewing, and approving the final version.

## CONFLICT OF INTEREST STATEMENT

The authors have no relevant financial or non‐financial interests to disclose.
